# Parent-provider paediatric literacy communication: A curriculum for future primary care providers

**DOI:** 10.1007/s40037-019-0503-8

**Published:** 2019-03-25

**Authors:** Tiffany Kindratt, Brittany Bernard, Jade Webb, Patti Pagels

**Affiliations:** 1 Department of Physician Assistant Studies, University of Texas Southwestern Medical Center, School of Health Professions, Dallas, TX USA; 2 Children’s Minnesota, Minneapolis, MN USA; 3 Questcare Partners, Dallas, TX USA; 4 Community Health Section, Department of Family and Community Medicine, University of Texas Southwestern Medical School, Dallas, TX USA

**Keywords:** Reach Out and Read, Objective structured clinical exam, Primary care, Early childhood literacy, Physician assistant, Postgraduate, Medical students, Paediatrics

## Abstract

**Background:**

Reach Out and Read promotes early literacy and school readiness by incorporating book delivery and anticipatory guidance into well-child visits. There is a need to train future healthcare providers in the knowledge and skills to communicate with parents/caregivers about early childhood literacy. We developed and evaluated a curriculum to improve learners’ knowledge, attitudes, and skills towards the incorporation of parent-provider literacy communication into well-child visits.

**Methods:**

Family medicine residents (*n* = 30), physician assistant students (*n* = 36), and medical students (*n* = 28) participated in a curriculum consisting of service learning, online didactic training, objective structured clinical exams (OSCEs) and a debriefing session. Standardized patients (SPs; 6 months to 5 years) and standardized patient caregivers were recruited and trained. Learners were evaluated on their abilities to offer books to patients, provide anticipatory guidance, and demonstrate parent-provider communication skills. Knowledge, attitudes, and satisfaction were collected pre- and post-curriculum.

**Results:**

Significant increases in total knowledge were observed after completing curriculum activities (*p* < 0.001). All attitudes improved after training (*p* < 0.05). All learners (100%) recommended that caregivers talk back and forth with their 6‑ to 12-month-old babies and make eye contact. Few (18.2%) learners recommended playing games like ‘peek-a-boo’ while reading. When caregivers evaluated learners’ basic parent-provider communication skills, all reported that the learners treated them with respect and used plain language.

**Discussion:**

Our curriculum extends beyond previous studies by measuring recommended books, anticipatory guidance, and communication skills using paediatric SPs and standardized patient caregivers. Curriculum activities can be tailored to best promote parent-provider literacy communication training in other programs.

**Electronic supplementary material:**

The online version of this article (10.1007/s40037-019-0503-8) contains supplementary material, which is available to authorized users.

## Background

Efforts have been made to improve patient-provider communication education in medical schools, residencies, and physician assistant programs [1]. Accrediting bodies require instruction in communication skills for an effective exchange of health information with patients [2, 3]. Limited training has focused on communication with parents/caregivers. The American Academy of Family Physicians recommends that the curriculum be designed to train future providers how to communicate with patients and caregivers and promote early literacy development at well-child visits [4].

Since 1989, Reach Out and Read has promoted early literacy in clinics in the United States through shared reading between parents and children. Evidence suggests that shared reading improves brain function, language, and school readiness [5]. Reach Out and Read promotes shared reading by distributing books to patients aged 6 months to 5 years, delivering anticipatory guidance to parents on reading aloud, and assessing developmental literacy milestones during well-child visits [6, 7]. A recent study demonstrated that by including Reach Out and Read in clinics, children were more receptive to reading, more likely to read with family and, ultimately, had improved language skills [8]. While the literature has expanded on the benefits of Reach Out and Read, limited research has focused on training providers. Studies have shown that residency training may improve knowledge, skills, and attitudes towards paediatric literacy concepts [9, 10]. However, these studies lacked simulated experiences where learners could practice communication skills with patients/caregivers and receive formative feedback. Objective structured clinical exams (OSCEs) have been used for over 40 years to provide formative feedback [11]. OSCEs utilize brief (5–10 min) clinical encounters with trained standardized patients (SPs). While OSCEs were developed for assessments of history and physical examinations, their use has expanded with the changing healthcare landscape. OSCEs are now utilized to measure patient-provider communication among patients from special populations (e. g. limited health literacy) [12], and to incorporate health information technology (e. g. mobile phone applications) [13] into patient encounters. There remains a gap in the literature on the use of OSCEs for training future providers how to communicate with paediatric patients and parents/caregivers. No studies have evaluated paediatric literacy training for physician assistant students, medical students, or interprofessional learners.

Our purpose was to develop and evaluate a curriculum to improve learners’ knowledge, attitudes, and skills towards paediatric literacy concepts. In this article, we present our findings and curriculum materials for use by other training programs.

## Evaluation of innovation

### Methods

Learners (family medicine residents *n* = 30; medical students *n* = 28; physician assistant students *n* = 36) participated from the University of Texas Southwestern Medical Center. Learners completed all or some curriculum activities as part of a community-based rotation (residents) [14], preclinical elective (medical students), or population health-related course (physician assistant students).

### Development and implementation

The curriculum included service learning, online didactic training, OSCE stations, and a classroom-based debriefing session [15] developed from previous research and curriculum recommendations [4, 9, 10, 16, 17]. Learners had opportunities for service learning at clinics embedded in local homeless shelters. Reach Out and Read programs began at the shelter for women and children in 2013 [17] and the domestic violence shelter in 2016. The shelter for women and children has a student-run clinic managed by medical and physician assistant students. Each session is staffed with four learners and one manager. Learners are supervised by the medical director, who is a certified physician assistant. In 2015, managers received lecture-based training on how to distribute books, discuss anticipatory guidance with parents, and document both in electronic medical records (EMRs). This training improved managers’ skills [17]. However, other volunteer learners did not receive training. Based on the growing number of volunteers and lack of training available, there was a need to implement a comprehensive parent-provider paediatric literacy curriculum. The training benefits patients and caregivers by equipping learners with knowledge and skills to provide anticipatory guidance, discuss reading, and provide books at no cost to homeless families.

The didactic curriculum was developed to expand the national Reach Out and Read training [16]. The online training included four modules: (1) benefits of Reach Out and Read; (2) ways to incorporate books in children’s lives; (3) teachable moments/techniques for clinical settings; and (4) research. The expanded training was developed so learners could: complete the training without being listed as an official provider for a designated Reach Out and Read clinic; obtain and complete trainings without internet access; and learn how to document book delivery and anticipatory guidance in EMRs.

OSCEs were developed and implemented based on their acceptability in our programs [12, 13] and ability to improve communication skills [11]. Paediatric standardized patients (SPs) and standardized patient caregivers were recruited and trained. Caregivers received training materials 2 weeks beforehand by email and 1 hour of in-person training prior to administering stations. Stations represented children accompanied by a mother, father, grandparent, or non-relative caregiver. Learners had 2 minutes to read a prompt on the door of each station instructing him/her to: give a book to the patient; provide anticipatory guidance; and practise basic parent-provider communication. Each learner completed two stations: (1) infants/toddlers (<2 years old) and (2) pre-schoolers (2–5 years old). OSCE station overviews, prompts, and caregivers scripts are provided in the online Supplementary Tables 1–5.

Learners reflected on their experiences with a certified physician assistant and the director of Reach Out and Read Texas during the debriefing session immediately following the OSCE stations. Learners shared service learning experiences and discussed how they would incorporate the knowledge and skills they learned into their future practice.

### Evaluations

Demographics were collected (gender, race/ethnicity) from program data sources. Outcomes were evaluated using three of Kirkpatrick’s levels of evaluation including (1) reaction, (2) learning, and (3) behaviour [18]. Learners received emails with a link to pre- and post-test surveys. Pre-test data were collected at the beginning of each course/rotation. Post-test data were collected immediately after completing the OSCE and debriefing session. Among those who did not complete the OSCE and debriefing session, post-tests were collected immediately after online training.

Learners answered four questions assessing their satisfaction with the curriculum activities. To measure changes in self-perceived knowledge and attitudes, learners completed nine pre- and post-test items using the same questions (see Tab. 1 for questions). Knowledge was measured with dichotomous variables (correct vs. incorrect). All pre- and post-knowledge questions were added together to create total pre- and post-knowledge scores. Attitudes were measured using 5‑point Likert scales (1 = strongly disagree; 5 = strongly agree).

**Table 1:** Demographics, knowledge^a^, attitudes^a^, and satisfaction of medical learners

**Demographics (*n*** **=** **94)**	***N*** **(%)**		
*Gender*			
Female	65 (69.1)		
Male	29 (30.9)		
*Race/Ethnicity*			
White/Caucasian	30 (31.9)		
Black/African American	3 (3.2)		
Hispanic	11 (11.7)		
Asian American	25 (26.6)		
Other/not reported	25 (26.6)		
*Medical learner group*			
Physician assistant students	36 (38.3)		
Medical students	28 (29.8)		
Family medicine residents	30 (31.9)		
**Pre- and Post-Test comparisons**	**Pre-test** **correct**	**Post-test** **correct**	
**(n** **=** **53)**	**n (%)**	**n (%)**	**P**
*Knowledge* ^*b*^			
1. Reach Out and Read serves over 5 million children annually in the US	50 (94.3)	46 (95.8)	0.0833
2. Watching Sesame Street is least likely to foster reading and writing	30 (56.6)	39 (81.3)	0.0075
3. Most children turn pages in board books by 18 months	48 (90.6)	45 (93.8)	0.4142
4. It is important to read a book word for word, even if very young	36 (67.9)	43 (89.6)	0.0075
5. Reach Out and Read books should be given to all children aged 12 and under *(answer: false—under 5 years)*	6 (11.3)	14 (29.2)	0.1655
	**Mean (SD)**	**Mean (SD)**	
Total knowledge	3.21 (0.93)	3.90 (0.69)	0.0002
*Attitudes* ^*c*^			
6. I feel comfortable assessing literacy during paediatric clinic visits	2.83 (0.81)	3.89 (0.52)	<0.0001
7. Parents are *(not)* offended by questions about literacy	3.15 (0.96)	3.74 (0.90)	0.0054
8. The clinic is an appropriate place to encourage literacy	4.12 (0.81)	4.57 (0.54)	0.0034
9. Literacy assessments and related anticipatory guidance tips are *(not)* only necessary when children are close to school age	2.90 (1.35)	4.38 (1.01)	<0.0001
*Post-curriculum satisfaction*	–	**Mean (SD)**	*–*
10. Clear objectives were provided	–	3.89 (0.73)	–
11. Information was provided that met my training needs	–	3.98 (0.68)	–
12. I can use this information to improve patient care	–	4.30 (0.63)	–
13. Overall, the training was very good	–	4.16 (0.67)	–

^a^Scale switched for questions 7 and 9 for analysis

^b^McNemar tests used for comparisons, significance level p < 0.05

^c^Wilcoxon signed-rank tests used for comparisons, significance level p < 0.05

To measure behaviour, caregivers scored learners (yes or no) on their ability to provide anticipatory guidance (e. g. name things), recommend age-appropriate books (e. g. board books), and demonstrate communication skills (e. g. introduced him/herself). OSCE scoresheets were developed based on Reach Out and Read’s ‘Milestones of Early Literacy Development Chart’ [16]. See online Supplemental Tables 1–5 for questions.

### Analysis

Data analysis was performed using STATA 14.0. Means and standard deviations were used to report satisfaction. McNemar tests were used to determine significant differences in pre- and post-test knowledge items. Wilcoxon signed-rank tests were used to determine significant differences between pre- and post-knowledge total scores. Wilcoxon tests were used to determine significant differences between pre- and post-attitudes. Frequencies and percentages were used to describe learners who demonstrated the expected behaviours during OSCE stations. Wilcoxon and chi-square tests were used to compare changes in attitudes, knowledge, and post-satisfaction between learners who did and did not participate in the OSCE and debriefing session.

The University of Texas Southwestern Medical Center’s Institutional Review Board deemed this project as non-human subjects research.

## Results

Tab. 1 reports demographics, and pre-, and post-test comparisons. Most learners were female (69%). Over half were white (32%) or Asian (27%) race/ethnicity. More were physician assistant students (38%) compared with residents (32%) or medical students (30%). All learners were required to complete the online training. There were 68 learners who gained service learning experiences at the homeless shelters, 53 who completed the pre- and post-tests, and 38 who completed the OSCE stations and debriefing session. Only two residents and no medical students were able to participate in the OSCEs and debriefing session.

Learners gained the most knowledge on the impact of technology and reading aloud on language development. Most (81%) learners identified that watching television shows like ‘Sesame Street’ was least likely to foster reading and writing after training compared with only 57% before training (*p* = 0.0075). Total knowledge scores were significantly higher post-curriculum (*p* < 0.0001). All attitudes improved after training. Learners were more likely to disagree with the statement ‘literacy assessments and related anticipatory guidance tips are only necessary when children are close to school age’ (*p* < 0.0001). Learners expressed their greatest level of satisfaction with their ability to use what they learned to improve patient care (mean = 4.30). No significant differences were found in knowledge, attitudes, or post-curriculum satisfaction between learners who did and did not participate in the OSCE stations and debriefing session.

Tab. 2 reports frequencies and percentages of learners who demonstrated the expected behaviour during OSCE stations. For 6‑ to 12-month-old SPs, all learners (100%) recommended that caregivers talk back and forth with their babies and make eye contact. Few (18.2%) recommended playing games like ‘peek-a-boo’ while reading. Most (92.6% = 12–24 months; 90.9% = 2–3 years) recommended caregivers use books in daily routines. When caregivers evaluated learners’ basic parent-provider communication skills, most learners introduced themselves when they entered the room (98.6%) and sat while talking to the SP and caregiver (92.1%).

**Table 2:** Frequency and percentages of medical learners who demonstrated the expected behaviour during the OSCE stations^a^

Station 1		Station 2		Station 3		Station 4		Station 5	
6–12 months (*n* = 11)		12–24 months (*n* = 27)		2–3 years (*n* = 11)		3–4 years (*n* = 14)		4–5 years (*n* = 13)	
	*n* (%)		*n* (%)		*n* (%)		*n* (%)		*n* (%)
*Anticipatory guidance*									
Talk back and forth with baby	11 (100)	Smile, answer when child speaks	27 (100)	Ask questions ‘what is that?’	9 (81.8)	Ask questions: ‘what’s next?’	13 (92.9)	Relate story to your child’s experiences	13 (100)
Make eye contact with baby	11 (100)	Let your child help turn the pages	21 (77.8)	Read same book multiple times	7 (63.6)	Point out letters, numbers	12 (85.7)	Let your child see you read	12 (92.3)
Cuddle, talk, sing, read, play	4 (36.4)	Use books in family routines	25 (92.6)	As you read, talk about the pictures	11 (100)	Point out pictures with same sounds	11 (78.6)	Ask your child to tell the story	12 (92.3)
Point at and name things	3 (27.3)	Use books to distract child waiting	19 (70.4)	Keep using books in daily routines	10 (90.9)	Make up stories about pictures	12 (85.7)	Encourage writing, drawing	13 (100)
Follow baby’s cues for ‘more’ or ‘stop’	2 (18.2)	Continue naming things	26 (96.3)	Let child choose book	7 (63.6)	Let child choose book	12 (85.7)	Point out letters in child’s name	4 (30.8)
Play games like ‘peek-a-boo’	2 (18.2)	**–**	**–**	**–**	**–**	**–**	**–**	Let child choose book	13 (100)
*Types of books*									
Board/cloth books	10 (90.9)	Board books	26 (96.3)	Rhyming books	7 (63.6)	Counting books	4 (28.6)	Fairy tales, legends	13 (100)
Books with baby Faces	11 (100)	Picture books that name things	27 (100)	Picture books that tell stories	10 (90.9)	Picture books that tell longer stories	10 (71.4)	Longer stories, fewer pictures	13 (100)
Nursery rhymes	1 (9.1)	Rhyming books	6 (22.2)	Search/find books	2 (18.2)	Alphabet books	4 (28.6)	**–**	**–**
*Parent-provider communication skills*									
Introduced him/herself	11 (100)	Introduced him/herself	26 (96.3)	Introduced him/herself	11 (100)	Introduced him/herself	14 (100)	Introduced him/herself	13 (100)
Sat while speaking	11 (100)	Sat while speaking	26 (96.3)	Sat while speaking	10 (90.9)	Sat while speaking	12 (85.7)	Sat while speaking	11 (84.6)
Spoke slowly	11 (100)	Spoke slowly	27 (100)	Spoke slowly	11 (100)	Spoke slowly	14 (100)	Spoke slowly	13 (100)
Used words caregiver knows	11 (100)	Used words caregiver knows	27 (100)	Used words caregiver knows	11 (100)	Used words caregiver knows	14 (100)	Used words caregiver knows	13 (100)
Treated caregiver/ patient with respect	11 (100)	Treated caregiver/ patient with respect	27 (100)	Treated caregiver/ patient with respect	11 (100)	Treated caregiver/ patient with respect	14 (100)	Treated caregiver/ patient with respect	13 (100)
Gave right amount of information for time allowed	11 (100)	Gave right amount of information for time allowed	26 (96.3)	Gave right amount of information for time allowed	11 (100)	Gave right amount of information for time allowed	12 (85.7)	Gave right amount of information for time allowed	13 (100)

^a^Due to complexities of medical school and residency scheduling, 42 physician assistant students, 2 residents (PGY2 on community medicine rotation) and no medical students participated in the OSCE stations

Residents completed stations for ages 12–24 months and 3–4 years

## Reflection and critical review

Our purpose was to develop and evaluate an innovative curriculum to improve interprofessional learners’ knowledge, attitudes, and skills in paediatric literacy concepts. We found significant improvements in knowledge and attitudes after completion of curriculum activities, which is similar to previous studies with residents [8, 9]. Our study extends beyond this research by incorporating interprofessional learners and using paediatric SPs and standardized patient caregivers. All learners demonstrated at least one basic communication skill during OSCE stations. These results are similar to previous research evaluating communication skills using OSCEs with adult patients in our program [13]. Although there were no significant differences in results among learners who did and did not participate in OSCE stations, this innovative component allows learners to gain first-hand, simulated experiences with paediatric patients and parents, which is often neglected in medical education. Most medical and physician assistant students had no prior experience in practising parent-provider communication.

Our study contributes to the literature by highlighting the importance of providers discussing shared reading and early childhood literacy with parents during well-child visits. Recent research has emphasized how Reach Out and Read can make children more receptive to reading, more likely to read with their families, more likely to reach developmental language milestones, and make parents more compliant to well-child visits [6, 8, 19]. While these studies have demonstrated improvements in patient outcomes, little emphasis is placed on training of providers. With more simulated training of future providers, the impact of Reach Out and Read could be even greater.

Strengths of our curriculum include the use of interprofessional learners and trained standardized patients/caregivers. A limitation was that not all learners completed all curriculum activities. Only 56.7% completed pre- and post-tests. However, this response rate is higher than previous studies evaluating online survey responses [20]. Due to the complexities of residency and medical school schedules, only two residents and no medical students were able to complete the OSCE and debriefing session. OSCE stations were held on one day. Future OSCEs should be designed to better suit residency and medical student schedules. Our OSCE stations were not specifically designed for homeless patients. The focus of our stations was on patients with minimal risk for language and developmental delays. This focus was due to learners’ limited exposure to paediatric patients. Future OSCEs could be modified to address barriers experienced by underserved populations. While our curriculum was only evaluated at one institution, the supplementary materials provided can be tailored for other programs interested in paediatric literacy training, regardless of formal participation in Reach Out and Read.

Learners who participated in all or parts of our curriculum demonstrated significant improvements in knowledge and attitudes towards paediatric literacy concepts. While we experienced several logistical challenges, we were able to create a greater awareness of the importance of paediatric literacy in different phases of medical education and offer service learning in homeless shelter clinics participating in Reach Out and Read. Our next steps are to evaluate the curriculum impact on communication with and reading behaviours among mothers and children at the shelters and identify ways to empower future providers to participate in Reach Out and Read or similar programs in their practices.

## Caption Electronic Supplementary Material

The supplementary material includes the OSCE station materials for all age groups. Training programs interested in re-creating our stations can use this material in three ways: 1) put the “learner prompt” on the door of each station; 2) provide the overview and script to each standardized patient caregiver at the beginning of each station; and 3) use the scoresheet to measure paediatric literacy and parent-provider communication skills for formative feedback purposes.
