# Supplementary material for: Parent-provider paediatric literacy communication: A curriculum for future primary care providers
Source: Perspect Med Educ. 2019 Mar 25;8(2):110–7. doi: 10.1007/s40037-019-0503-8 (PMC6468016; doi:10.1007/s40037-019-0503-8)
Supplement: Supplementary file 1 — The supplementary material includes the OSCE station materials for all age groups. Training programs interested in re-creating our stations can use this material in three ways: 1) put the “learner prompt” on the door of each station; 2) provide the overview and script to each standardized patient caregiver at the beginning of each station; and 3) use the scoresheet to measure paediatric literacy and parent-provider communication skills for formative feedback purposes. [file 40037_2019_503_MOESM1_ESM.docx]

**Supplemental Table 1: 6-12 Months OSCE Station Overview, Prompts, Script and Scoresheet.**

| **6-12 Months Station Overview and Medical Learner Prompt for Door Sign** | | |
| --- | --- | --- |
| You will have 5 minutes to complete this station. This is a 6-month old patient and his grandmother. Enter the room to talk to the parent/ patient about literacy. Using the information you have learned about the Reach Out and Read program:   - Educate the parent/caregiver on what they can do to promote literacy in the child; - Give a book to the patient; - Provide anticipatory guidance based on the child’s developmental stage; - Practice your basic communication skills. | | |
| **Standardized Patient Caregiver (SPC) Overview and Script** | | |
| You have come to a well-child visit today with your pediatric patient. The medical learner will enter the room to discuss the importance of literacy before conducting a well-child exam.  Explain to the health care provider that you read to your grandchild occasionally, but you don’t really understand why it is important at such a young age when their attention span is limited anyway. You spend more time with him playing outside. You are a busy professional with work and volunteer obligations and do not have set routines for your child because your work schedule changes often. | | |
| **SPC Scoresheet** | | |
| **Scoresheet Instructions:** Place a checkmark “✓” in the “yes” or “no” boxes based on how the resident performed the assigned task. Count the total number of ✓s in the “Yes” Column at the end. If you do not have time to calculate the total score, you can leave it blank. Residents will receive 1 point for “Yes” and 0 points for “No” responses. | | |
|  | **Yes *(1 point)*** | **No *(0 points)*** |
| **The medical learner provided the following** ***anticipatory guidance***: | | |
| Talk back and forth with baby |  |  |
| Make eye contact with baby |  |  |
| Cuddle, talk, sing, read, play |  |  |
| Point at and name things |  |  |
| Follow baby’s cues for “more” or “stop” |  |  |
| Play games such as “peek-a-boo” |  |  |
| **The medical learner advised me that the following *types of* *books* as best for the child:** | | |
| Board/cloth books |  |  |
| Books with baby faces |  |  |
| Nursery rhymes |  |  |
| **The medical learner demonstrated the following *communication skills*:** | | |
| Introduced him/herself |  |  |
| Sat while speaking |  |  |
| Spoke slowly |  |  |
| Used words caregiver knows |  |  |
| Treated caregiver/patient with respect |  |  |
| Gave right amount of information for time allowed |  |  |
| **Total Scores (0 to 15 possible points):** |  | |
| **Open Ended:** Please identify ***strengths and areas of improvement:*** | | |

**Supplemental Table 2: 12-24 Months OSCE Station Overview, Prompts, Script and Scoresheet.**

| **12-24 Months Station Overview and Medical Learner Prompt for Door Sign** | | |
| --- | --- | --- |
| You will have 5 minutes to complete this station. This is an 18-24 month old patient and her mother. The well-child history & exam have already been completed and you will now re-enter the room to talk to the parent/patient about literacy. Using the information you have learned about the Reach Out and Read program:   - Educate the parent/caregiver on what they can do to promote literacy in the child; - Give a book to the patient; - Provide anticipatory guidance based on the child’s developmental stage; and - Practice your basic communication skills. | | |
| **Standardized Patient Caregiver (SPC) Overview and Script** | | |
| You have come to a well-child visit today with your ~18 month old patient. The medical learner has completed his/her exam of the patient and has stepped out of the room briefly. He/She will enter the room to discuss the importance of literacy. Explain to the health care provider that you read to your child occasionally, but you don’t really understand why it is important at such a young age, when their attention span is limited anyway. You are a busy professional and your child does not have set routines because your work schedule changes. | | |
| **SPC Scoresheet** | | |
| **Scoresheet Instructions:** Place a checkmark “✓” in the “yes” or “no” boxes based on how the resident performed the assigned task. Count the total number of ✓s in the “Yes” Column at the end. If you do not have time to calculate the total score, you can leave it blank. Residents will receive 1 point for “Yes” and 0 points for “No” responses. | | |
|  | **Yes *(1 point)*** | **No *(0 points)*** |
| **The medical learner provided the following** ***anticipatory guidance***: | | |
| Smile & answer when your child speaks or points |  |  |
| Let your child help turn the pages |  |  |
| Use books in family routines (i.e. playtime, bedtime) |  |  |
| Use books to calm or distract your child while waiting |  |  |
| Name things (ball, baby, dog, etc) |  |  |
| **The medical learner advised me that the following *types of* *books* as best for the child:** | | |
| Board books |  |  |
| Rhyming books |  |  |
| Picture books / books that name things |  |  |
| **The medical learner demonstrated the following *communication skills*:** | | |
| Introduced him/herself |  |  |
| Sat while speaking |  |  |
| Spoke slowly |  |  |
| Used words caregiver knows |  |  |
| Treated caregiver/patient with respect |  |  |
| Gave right amount of information for time allowed |  |  |
| **Total Scores (0 to 14 possible points):** |  | |
| **Open Ended:** Please identify ***strengths and areas of improvement:*** | | |

**Supplemental Table 3: 2-3 Years OSCE Station Overview, Prompts, Script and Scoresheet.**

| **2-3 Years Station Overview and Medical Learner Prompt for Door Sign** | | |
| --- | --- | --- |
| You will have 5 minutes to complete this station. This is a 2 year old patient and her mother. The well-child history & exam have already been completed and you will now re-enter the room to talk to the parent/patient about literacy. Using the information you have learned about the Reach Out and Read program:   - Educate the parent/caregiver on what they can do to promote literacy in the child; - Give a book to the patient; - Provide anticipatory guidance based on the child’s developmental stage; and - Practice your basic communication skills. | | |
| **Standardized Patient Caregiver (SPC) Overview and Script** | | |
| You have come to a well-child visit today with your 2 year old patient. The medical learner has completed his/her exam of the patient and has stepped out of the room briefly. He/She will enter the room to discuss the importance of literacy. Explain to the health care provider that you read at home if your child wants to read, but you do not have a set routine. Tell the provider that you did not read with your parents when you were a kid and don’t see the need to make an effort to do so now. Tell the provider that you pay “good money” for your child to learn to read in pre-school and that should prepare them for kindergarten/elementary school. | | |
| **SPC Scoresheet** | | |
| **Scoresheet Instructions:** Place a checkmark “✓” in the “yes” or “no” boxes based on how the resident performed the assigned task. Count the total number of ✓s in the “Yes” Column at the end. If you do not have time to calculate the total score, you can leave it blank. Residents will receive 1 point for “Yes” and 0 points for “No” responses. | | |
|  | **Yes *(1 point)*** | **No *(0 points)*** |
| **The medical learner provided the following** ***anticipatory guidance***: | | |
| Ask questions like "where's the dog?" or "what is that?" |  |  |
| Be willing to read the same book again & again |  |  |
| As you read, talk about the pictures |  |  |
| Keep using books in daily routines |  |  |
| Let your child choose which book to read. |  |  |
| **The medical learner advised me that the following *types of* *books* as best for the child:** | | |
| Rhyming books |  |  |
| Picture books that tell stories |  |  |
| Search and find books |  |  |
| **The medical learner demonstrated the following *communication skills*:** | | |
| Introduced him/herself |  |  |
| Sat while speaking |  |  |
| Spoke slowly |  |  |
| Used words caregiver knows |  |  |
| Treated caregiver/patient with respect |  |  |
| Gave right amount of information for time allowed |  |  |
| **Total Scores (0 to 14 possible points):** |  | |
| **Open Ended:** Please identify ***strengths and areas of improvement:*** | | |

**Supplemental Table 4: 3-4 Years OSCE Station Overview, Prompts, Script and Scoresheet.**

| **3-4 Years Station Overview and Medical Learner Prompt for Door Sign** | | |
| --- | --- | --- |
| You will have 5 minutes to complete this station. This is a 3 year old patient and his caregiver. The well-child history & exam have already been completed and you will now re-enter the room to talk to the patient/caregiver about literacy. Using the information you have learned about the Reach Out and Read program:   - Educate the parent/caregiver on what they can do to promote literacy in the child; - Give a book to the patient; - Provide anticipatory guidance based on the child’s developmental stage; and - Practice your basic communication skills. | | |
| **Standardized Patient Caregiver (SPC) Overview and Script** | | |
| You have come to a well-child visit today with your 3 year old patient. The medical learner has completed his/her exam of the patient and has stepped out of the room briefly. He/She will enter the room to discuss the importance of literacy. Explain to the health care provider that you read at home if your child wants to read, but you do not have a set routine. Tell the provider that you did not read with your parents when you were a kid and don’t see the need to make an effort to do so now. Tell the provider that you pay “good money” for your child to learn to read in pre-school and that should prepare them for kindergarten/elementary school. | | |
| **SPC Scoresheet** | | |
| **Scoresheet Instructions:** Place a checkmark “✓” in the “yes” or “no” boxes based on how the resident performed the assigned task. Count the total number of ✓s in the “Yes” Column at the end. If you do not have time to calculate the total score, you can leave it blank. Residents will receive 1 point for “Yes” and 0 points for “No” responses. | | |
|  | **Yes *(1 point)*** | **No *(0 points)*** |
| **The medical learner provided the following** ***anticipatory guidance***: | | |
| Ask "what happens next?" in familiar stories |  |  |
| Point out letters, numbers |  |  |
| Point out words & pictures that begin with same sounds |  |  |
| Together, make up stories about pictures |  |  |
| Let your child choose which book to read. |  |  |
| **The medical learner advised me that the following *types of* *books* as best for the child:** | | |
| Picture books that tell longer stories |  |  |
| Counting books |  |  |
| Alphabet books |  |  |
| **The medical learner demonstrated the following *communication skills*:** | | |
| Introduced him/herself |  |  |
| Sat while speaking |  |  |
| Spoke slowly |  |  |
| Used words caregiver knows |  |  |
| Treated caregiver/patient with respect |  |  |
| Gave right amount of information for time allowed |  |  |
| **Total Scores (0 to 14 possible points):** |  | |
| **Open Ended:** Please identify ***strengths and areas of improvement:*** | | |

**Supplemental Table 5: 4-5 Years OSCE Station Overview, Prompts, Script and Scoresheet.**

| **4-5 Years Station Overview and Medical Learner Prompt for Door Sign** | | |
| --- | --- | --- |
| You will have 5 minutes to complete this station. You are seeing a 4 & 5 year old patients and their father. The well-child history & exams have already been completed back-to-back and you will now re-enter the room to talk to the patient/caregiver about literacy. Using the information you have learned about the Reach Out and Read program:   - Educate the parent/caregiver on what they can do to promote literacy in the child; - Give a book to the patient; - Provide anticipatory guidance based on the child’s developmental stage; and - Practice your basic communication skills. | | |
| **Standardized Patient Caregiver (SPC) Overview and Script** | | |
| You have come to well-child visits today with your 4 & 5 year old children. The medical learner has completed their exams and has stepped out of the room briefly. He/She will enter the room to discuss the importance of literacy. Explain to the health care provider that you read at home if your children want to read, but with two of them so close in age, you do not have set routines. Tell the provider that you did not read with your parents when you were a kid and don’t see the need to make an effort now. Tell the provider that your children learn to read in pre-school and that should prepare them for kindergarten/elementary school. | | |
| **SPC Scoresheet** | | |
| **Scoresheet Instructions:** Place a checkmark “✓” in the “yes” or “no” boxes based on how the resident performed the assigned task. Count the total number of ✓s in the “Yes” Column at the end. If you do not have time to calculate the total score, you can leave it blank. Residents will receive 1 point for “Yes” and 0 points for “No” responses. | | |
|  | **Yes *(1 point)*** | **No *(0 points)*** |
| **The medical learner provided the following** ***anticipatory guidance***: | | |
| Relate the story to your child's own experiences |  |  |
| Let your child see *you* read |  |  |
| Ask your child to tell the story |  |  |
| Encourage writing, drawing |  |  |
| Point out the letters in your child's name |  |  |
| Let your child choose which book to read |  |  |
| **The medical learner advised me that the following *types of* *books* as best for the child:** | | |
| Fairy tales/legends |  |  |
| Books with longer stories, fewer pictures |  |  |
| **The medical learner demonstrated the following *communication skills*:** | | |
| Introduced him/herself |  |  |
| Sat while speaking |  |  |
| Spoke slowly |  |  |
| Used words caregiver knows |  |  |
| Treated caregiver/patient with respect |  |  |
| Gave right amount of information for time allowed |  |  |
| **Total Scores (0 to 14 possible points):** |  | |
| **Open Ended:** Please identify ***strengths and areas of improvement:*** | | |
